# Species diversity of freshwater shrimp in Henan Province, China, based on morphological characters and *COI* mitochondrial gene

**DOI:** 10.1002/ece3.7855

**Published:** 2021-07-13

**Authors:** Chuan‐jiang Zhou, Meng‐xia Feng, Yong‐tao Tang, Chang‐xing Yang, Xiao‐lin Meng, Guo‐xing Nie

**Affiliations:** ^1^ College of Fisheries Engineering Technology Research Center of Henan Province for Aquatic Animal Cultivation Engineering Lab of Henan Province for Aquatic Animal Disease Control Henan Normal University Xinxiang China

**Keywords:** *COI*, freshwater shrimps, species delimitation, species diversity

## Abstract

Freshwater shrimp are a rich species group, with a long and problematic taxonomic history attributed to their wide distribution and similar morphological characteristics. Shrimp diversity and species identification are important cornerstones for fisheries management. However, identification based on morphological characteristics is a difficult task for a nonspecialist. Abundant freshwater shrimp species are distributed in the waters of Henan Province, but investigations of freshwater shrimp are limited in this region, especially concerning molecular features. Here, we combined morphology and DNA barcodes to reveal the species diversity of freshwater shrimp in Henan province. A total of 1,200 freshwater shrimp samples were collected from 46 sampling sites, and 222 samples were chosen for further microscopic examination and molecular delimitation. We used tree‐based methods (NJ, ML, and bPTP) and distance‐based methods (estimation of the paired genetic distances and ABGD) to delimit species. The results showed that there were nine morphospecies based on morphological characteristics; all could effectively be defined by molecular methods, among which bPTP and ABGD defined 13 and 8 MOTUs, respectively. The estimation of the paired genetic distances of K2P and the p‐distances had similar results. Mean K2P distances and p‐distances within species were both equal to 1.2%. The maximum intraspecific genetic distances of all species were less than 2%, with the exception of *Palaemon modestus* and *M. maculatum*. Various analyses have shown that *P. modestus* and *M. maculatum* have a large genetic differentiation, which may indicate the existence of cryptic species. By contrast, DNA barcoding could unambiguously discriminate 13 species and detect cryptic diversity. Our results demonstrate the high efficiency of DNA barcoding to delimit freshwater shrimp diversity and detect the presence of cryptic species.

## Graphical abstract

This study used a combination of morphology and molecular methods to comprehensively study the phylogenetic relationship and species diversity of freshwater shrimp in Henan Province.

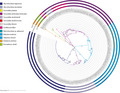

## 1 INTRODUCTION

Freshwater shrimp (Decapoda: Caridea: Caridean) are a highly species‐rich group with a long taxonomic history. However, the taxonomic status of these shellfish is controversial (De Grave et al., 2014; Martin & Davis, 2001). There are about 770–800 Caridea species in freshwater habitats, accounting for about one‐fifth of the described shrimp species (De Grave et al., 2015). At present, freshwater shrimp exist in seven Caridea families (De Grave et al., 2014). The two families Atyidae and Palaemonidae dominate, comprising 443 and 300 species, respectively, and accounting for 97.4% of freshwater shrimp species (De Grave et al., 2015). Shrimp are an important component of biodiversity, as they provide a source of animal protein for people. In addition, freshwater shrimp have significant economic and nutritional value and research significance (Holthuis, 1980; New & Nair, 2012). At present, Jamaica (Hunte, 1978), Japan (Suzuki et al., 1993), Myanmar (Cai & Ng, 2002), China (Li et al., 2007; Liang, 2004), and many Chinese provinces (Deng & Wu, 1997; Zheng, 1989; Zhu & Miao, 1990) have carried out studies on the species diversity of freshwater shrimp, but most of the early studies were based on traditional morphological characteristics. The molecular methods have been gradually applied to research on the diversity of freshwater shrimp in recent years (De Grave et al., 2008; Makombu et al., 2019; Mar et al., 2018; New & Nair, 2012).

Studying species diversity is basic to biological research, but it is also a huge challenge and a harsh burden (Hebert, Cywinska, et al., 2003). As the main method of species diversity research, traditional morphological identification has high requirements and restrictions on samples and researchers, and the identification results are affected by both subjective and objective factors (Carvalho et al., 2011; Hebert, Ratnasingham, 2003; Shen et al., 2016). Since the early 2000s, DNA barcode technology has rapidly developed and has gradually become one of the main methods for biological identification (Hebert, Ratnasingham, et al., 2003). Compared with traditional morphological identification, barcode technology has many advantages. First, DNA is more stable than morphological characteristics, because DNA characters are constant throughout development. However, morphological characteristics vary with age, developmental stage, environment, and other factors. For example, molecular identification of deformed and underdeveloped shrimp larvae has absolute advantages over morphological identification (Burghart et al., 2014; Lee & Kim, 2014). Second, one can obtain sample DNA through some small parts of tissues, secretions, and even an organism's living environment (Pont et al., 2018), which reduces the requirements of sampling (Chang et al., 2016). More importantly, DNA barcoding is easy to operate, fast, and efficient. Samples can be identified in batches, and the method requires less professional knowledge (Takahara et al., 2013; Tinacci et al., 2018). With the implementation of the Barcoding of Life project, DNA barcodes have been widely recognized as a basic tool for species identification, and the mitochondrial gene cytochrome *c* oxidase I (*COI*) serves as the core of the global animal biometric system could effectively distinguish species of Crustacea (Costa et al., 2007; Hebert, Cywinska, et al., 2003; Hebert, Ratnasingham, et al., 2003).

In the era of high‐throughput sequencing, there is the probability of tentative, incorrect, or low‐quality sequences being submitted to databases (Wong et al., 2011). Compared with the commonly used barcode databases GenBank (National Center for Biotechnology Information, NCBI), DDBJ (DNA Data Bank of Japan), and EMBL‐EBI (The European Molecular Biology Laboratory‐European Bioinformatics Institute), the BOLD (the Barcode of Life Database) database conducts strict review and screening of submitted data, and thus, it is relatively more accurate and applicable (Macher et al., 2017; Wang et al., 2009). In addition, with the acquisition of a large number of barcodes, there has been growing use of molecular approaches for species delimitation; this has improved the accuracy of species identification (Hebert & Gregory, 2005; Luo et al., 2018). At present, tree‐based methods, distance‐based methods, and character‐based methods are commonly used in DNA‐barcoding studies (Birch et al., 2017). The combined use of multiple methods will make the results of species delimitation more objective and comprehensive (Schlick‐Steiner et al., 2010). Therefore, as many different types of molecular methods as possible should be used for comprehensive species identification.

Henan province is located inland and harbors four major river systems, the Yellow River, the Yangtze River, the Huaihe River, and the Haihe River. Our investigation of fisheries in Henan Province has shown that there are abundant fishery resources, but research on the province's freshwater shrimp is relatively scarce, and thus, the status of freshwater shrimp species diversity is relatively unknown. To date, eight species of shrimp have been reported; surveys have used traditional morphological recognition methods to identify 352 samples and describe eight species from 15 sampling points (Wang, 1989). In view of the above, it is important to enrich shrimp‐related research in Henan province in order to append the list of shrimp species and to assess the biodiversity in this area.

Combining molecular and morphological evidence in taxonomy is advocated (DeSalle et al., 2005; Miralles & Vences, 2013), so both morphological identification and molecular definitions have been used for species identification of freshwater shrimp that covered most of rivers in Henan Province, China, in our study. In order to obtain more objective species identification, multiple methods were employed. The main aims of this study were (a) to assess the shrimp diversity based on morphological features; (b) to build a reference DNA‐barcoding library for these morphological species, and (c) to detect whether cryptic diversity occurred in shrimp in the province. Our study will provide helpful information for future conservation and fisheries management of the shrimp in Henan province.

## 2 MATERIALS AND METHODS

### 2.1 Ethics statement

The study conformed to the National Institutes of Health Guide for the Care and Use of Laboratory Animals (NIH Publication No. 85‐23, 1996) (2011).

### 2.2 Sample collection

**FIGURE 1:**
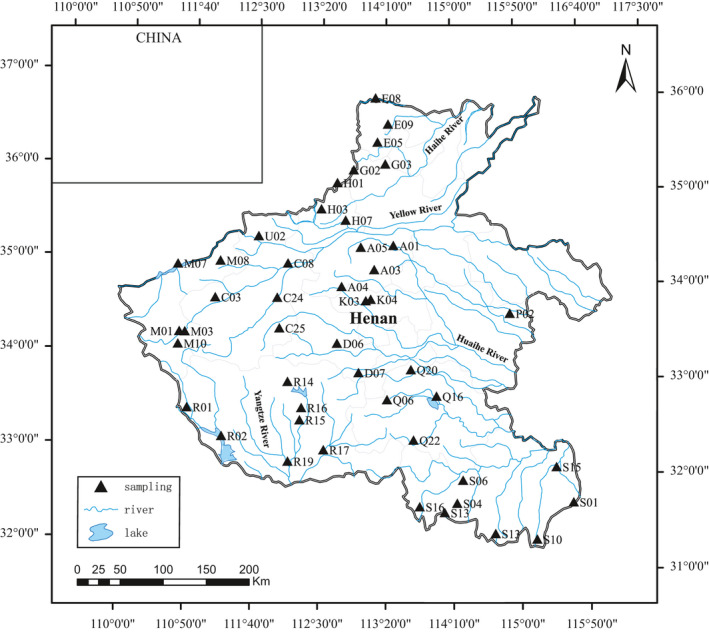
Sampling sites of freshwater shrimp in Henan province

A total of 46 sampling sites were covered in this survey for collecting freshwater shrimp (Figure 1). The sampling sites covered the main streams and tributaries of the four major rivers (i.e., the Yangtze River, the Huaihe River, the Yellow River, and the Haihe River) of the province (Table S1). In this study, about 1,200 samples representing nine species, six genera, and four families were collected. Most of the shrimp were collected by shrimp traps, but many individuals were obtained from markets. The samples were preserved in 95% ethanol for subsequent morphological observation and molecular identification. All voucher specimens were stored in the Fisheries College of Henan Normal University.

### 2.3 Morphological identification

Morphological identification was mainly classified in situ by visual inspection in the field, and then detailed morphological identification and classification were conducted in the laboratory by stereomicroscope microscopic examination. All samples were taxonomically classified based on the distinguishing morphological characters of the male collected specimens according to Liu (1955), Liang (2004), and Li et al. (2007).

### 2.4 DNA extraction, amplification, and sequencing

According to the results of morphological identification, multiple representative individuals of each taxonomic group were selected for abdominal muscle sampling. The obtained tissue samples were immediately stored in 95% ethanol and numbered for DNA extraction. To ensure the coverage of each species, individuals with moderate body size were selected as far as possible for EP tube preservation and numbering, and the larger individuals were marked with winding coils.

Genomic DNA was extracted by phenol‐chloroform (Sambrook & Russel, 2001) from muscle tissue (0.1–0.15 g) and verified using 1.0% agarose gel electrophoresis.

The amplification of the *COI* gene was carried out by polymerase chain reaction (PCR). A 632 bp fragment was amplified using the forward primer (LCO1490: 5′‐GGTCAACAAATCA TAAAGATATTGG‐3′) and reverse primer (HCO2198: 5′‐TAAACTTCAGGGTGACCAAAAAA TCA‐3′) (Folmer et al., 1994). PCRs were performed in a total volume of 50 μl containing 50–100 ng DNA template, 5 µl of 10× PCR buffer, 1.5 mmol/L of MgCl_2_, 0.2 mmol/L of each dNTP, 2 unit (U) of Taq polymerase, and 0.2 µmol/L of each primer. Thermal cycling began with one cycle of pre‐denaturation at 94°C for 5 min, 35 cycles of denaturation at 94°C for 30 s, annealing at 50°C for 45 s, extension at 72°C for 45 s, and a final extension holding at 72°C for 7 min (Feng et al., 2008). The PCR products were separated by electrophoresis on 1.0% agarose gels.

Primer synthesis and DNA sequencing were conducted by commercial companies. Among the 222 specimens, 141 were sequenced in one direction (63.51%), and the other specimens were two‐way sequenced. Except for the sequences obtained from the genomic DNA in this study, the other *COI* sequences were obtained from GenBank for comparative analyses (Table S2).

### 2.5 Sequencing analysis

The chromatogram inspection, alignment, and calibration of the original sequences used SeqMan (Swindell & Plasterer, 1997) of the DNASTAR Lasergene software package (DNASTAR, Inc., Madison, Wisconsin, USA). BioEdit v 7.0.9 (Tippmann, 2004) was used to align and shear sequences.

In this study, traditional morphological identification and a variety of different molecular methods were used for comprehensive analysis and species delimitation. Due to the uneven sampling and the differences in effective population sizes of species (Blair & Bryson, 2017), we chose Automatic Barcode Gap Discovery (ABGD) and Poisson tree processes (PTP) for quantifying and delimiting taxonomic diversity. The specific analysis is described below.

### 2.6 Distance‐based approaches

Given that previous studies showed that the use of the Kimura‐2‐parameter (K2P) model in DNA‐barcoding studies is poorly justified, but no more suitable model has been derived at present; therefore, in order to obviate the requirement for model correction in DNA barcoding, a p‐distance model was used in our analysis and calculations, while the K2P model was also used (Srivathsan & Meier, 2012; Collins et al., 2012). The K2P and p‐distance models were used to construct a neighbor‐joining tree and to calculate the pairwise genetic distances using MEGA 7.0 (Kumar et al., 2016). The haplotype diversity and nucleotide diversity of *COI* sequences were calculated using DnaSP 5.0 (Librado & Rozas, 2009). Then, ML tree analysis was implemented using RaxmlGUI (Stamatakis, 2014) with the default parameters and 1,000 replications. In all trees, bootstrap values below 70% are not shown.

Each sequence was selected for further species confirmation by the IDENTIFICATION of BOLD and the BLAST of NCBI to evaluate the accuracy of the morphological identification and to obtain reference sequences with high relative similarity. In the selection of similar sequences, we have defined 97% as a relatively loose standard to indicate potential species identification (Wong & Hanner, 2008).

In this study, a total 42 *COI* sequences with high similarity were obtained by aligning from GenBank. *Gammarus pisinnus* (GenBank accession number: KF824592) was selected as outgroup. All novel sequences obtained in this study were submitted to GenBank, and their accession numbers are provided in the Electronic Appendix (Table S2).

In addition, ABGD analysis was implemented on the website (https://bioinfo.mnhn.fr/abi/public/abgd/abgdweb.html), using K80, relative gap width (*X* = 1.5), and the remaining parameters as default values (Puillandre et al., 2012).

### 2.7 Tree‐based approach

A large number of tests have shown that PTP is superior to GMYC (Generalized mixed Yule‐coalescent) on simulated data, and the results are comparable to GMYC on real datasets. Meanwhile, PTP requires less data and only a simple phylogenetic tree (Luo et al., 2018; Zhang et al., 2013). Therefore, in this study, we chose PTP analysis to assist in species definition. PTP can delimit species based on the Phylogenetic Species Concept. Therefore, the entities output by PTP are in theory species. Bayesian Poisson tree process (bPTP) analysis was run on the web server (https://species.h‐its.org/ptp) with 100,000 MCMC generations, and other parameters as default values (Stamatakis 2006).

## 3 RESULTS

### 3.1 Morphological identification

**FIGURE 2:**
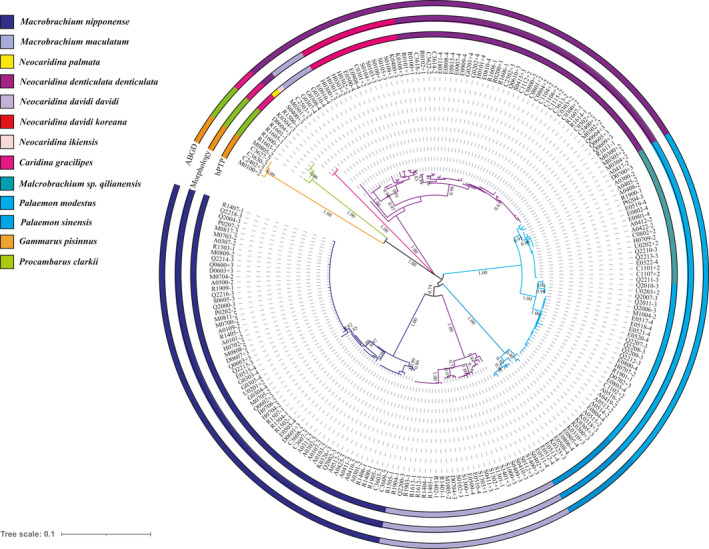
Classification of freshwater shrimp with molecular methods

According to the morphological characteristics obtained by the in situ visual examination and stereomicroscope microscopic examination, the 1,200 samples collected in Henan province comprised nine species from two orders, four families, and six genera. The detailed identification results are shown in Figure 2. The individual morphological variation of *N. denticulate*, *Neocaridina davidi* (Bouvier, 1904), *M. maculatum*, and *M. nipponense* is obvious, especially the morphology of their rostrums (Figure S1). Morphological observation results showed that some individuals of the above species had varying degrees of differences and changes in the length, shape, and tooth form of their rostrums. Those morphological changes were at times inconsistent with the descriptions in the literature (Li et al., 2007; Liang, 2004; Liu, 1955), even exceeding the range of variation of those described species. In addition, consistent with the general distribution trend of freshwater shrimp, specimens in this province mainly belonged to *Macrobrachium* and *Neocaridina*. Among these species, *M. nipponense,* *N. denticulate,* and *N. davidi* were dominant species in Henan Province.

### 3.2 Molecular delimitation

#### 3.2.1 Database search

In general, our morphological identification results matched the BLASTN annotations of the NCBI and BOLD databases, with at least 97% identities (Wong & Hanner, 2008). According to the identification results of Species Level Barcode Records of the BOLD reference sequence library, *P. clarkii*, *N. denticulate*, *Neocaridina* *davidi*, *C. gracilipes*, *M. maculatum,* and *M. nipponense* were identified to the species level. The identification results were relatively reliable, suggesting effective identification of the species. The identities of *Macrobrachium* sp. “*qilianensis*” and *P. modestus* were all greater than 98%, but the search results showed that the sequence identities between *P. modestus* and the three unpublished *M*. sp. “*qilianensis*” in the library were also high (at times having the highest identities). At the same time, in the retrieval of *M*. sp. “*qilianensis*,” the identities of this and two unpublished *P. modestus* were also relatively high. After verification, the above *M*. sp. “*qilianensis*” (Accession: FJ958200, FJ958201) was sourced from GenBank and was found to be a direct and unpublished submission by Cheng (2009). However, there was no corresponding morphological description, and species identification of *M*. sp. “*qilianensis*” was found in his study (Zhang et al., 2009). In addition, the search results for *G. pisinnus* were only 96%–98%, and the identities were slightly lower; the search results for *P. sinensis* showed no corresponding records. The search results for Species Level Barcode Records were similar to the search results for Species Level Barcode Records, and these will not be repeated here; the NCBI search showed a trend similar to the BOLD results, while the same species sequence was retrieved for *P. sinensis* (MK994929, MK994930).

#### 3.2.2 Species delimitation

**FIGURE 3:**
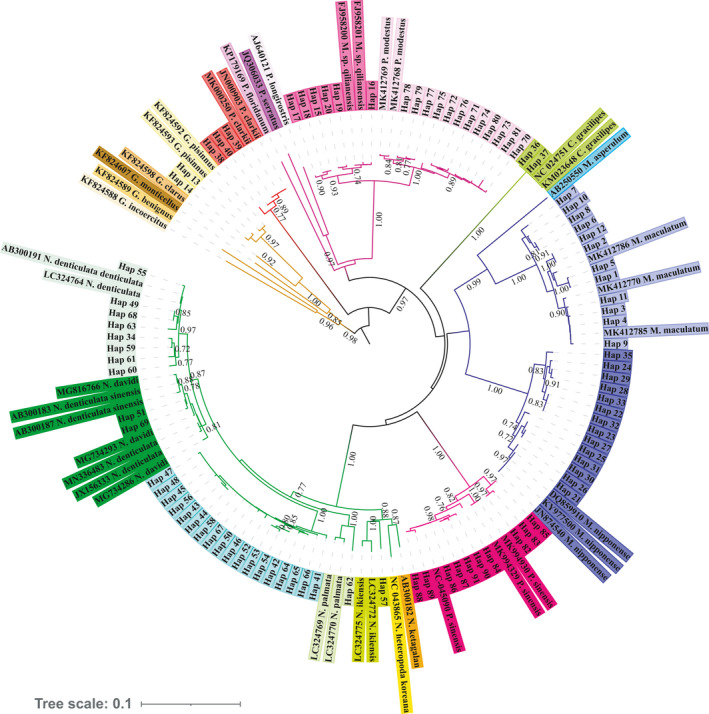
The haplotype Maximum likelihood phylogenetic tree of the 222 obtained sequences

The NJ tree based on the p‐distance model is not shown because of the same topology as for the K2P model. The NJ phylogenetic analysis showed that freshwater shrimp in Henan Province formed a total of 13 monophyletic clades, with *M. maculatum*, *Neocaridina* *davidi,* and *Palaemon modestus* further subdivided into no less than one clade each. The results showed that *M*. sp. “*qilianensis*” and *P. modestus* are sister clades, and *N. davidi* and *N. davidi koreana* are sister clades. The NJ phylogenetic analysis revealed that all 222 sequences were divided into at least 13 MOTUs (molecular operational taxonomic units) (Figure 2). The analyses of haplotype diversity and nucleotide diversity (Table 1) showed that the 222 sequences obtained were divided into 91 haplotypes, with widely distributed species such as *M. nipponensis*, *Palaemon modestus,* and *M. maculatum* having greater genetic differentiation (Figure 3).

**TABLE 1:** Information concerning freshwater shrimp sequences of this study

Order	Family	Genus	Species	Haplotype diversity (Hd)	Nucleotide diversity (*p*)	Reference sequence GenBank accession no.	GenBank Accession no. (sample number)
Decapoda	Cambaridae	*Procambarus*	*P. clarkii*	0.70000	0.00634	MK000250, JN000903	MW069604–MW069608
	Palaemonidae	*Macrobrachium*	*M. maculatum*	0.86462	0.02192	MK412770, MK412785, MK412786	MW069488–MW069513
			*M. nipponensis*	0.73189	0.01237	KY977500, JN874540, DQ859910	MW069539–MW069600
			*M. asperulum*	/	/	AB250550	/
		*Palaemon*	*P. sinensis*	0.98182	0.01559	MK994329, MK994330, NC‐045090	MW069699–MW069709
			*Palaemon modestus* (***M. sp. qilianensis***)	0.89952	0.02466	MK412768, MK412769, **FJ958200, FJ958201**	MW069518–MW069538; MW069673–MW069698
			*P. modestus*	0.90769	0.00841	MK412768, MK412769	MW069518–MW069538
			***M. sp. qilianensis***	0.63810	0.00519	**FJ958200, FJ958201**	MW069673–MW069698
			*P. serratus*	/	/	JQ306033	/
			*P. longirostris*	/	/	AJ640121	/
			*P. floridanum*	/	/	KP179169	/
	Atyidae	*Neocaridina*	*N. denticulate*	0.92248	0.00595	/	MW069628–MW069670
			*Neocaridina davidi* **(*N.denticulata sinensis*)**	0.91228	0.01585	MG734286, MG734293, MG816766, MN336483, JX156333, AB300187, AB300183, LC324764, AB300191	**MW069609–MW069627**
			*N. davidi davidi*	0.40000	0.00063	LC324764, AB300191	MW069623–MW069627
			*N. davidi korea*	0.90110	0.01109	MG734286, MG816766, MN336483, JX156333, AB300187, AB300183	MW069609–MW069622
			***N. ikiensis***	N/C	N/C	LC324772, LC324775	MW069671
			***N. palmata***	N/C	N/C	LC324769, LC324770	MW069672
			*N. ketagalan*	/	/	AB300182	/
		*Caridina*	*C. gracilipes*	0.66667	0.00106	KM023648, NC024751	MW069601–MW069603
Amphipoda	Gammaridae	*Gammarus*	*G. pisinnus*	0.50000	0.00317	KF824592, KF824593	MW069514–MW069517
			*G. clarus*	/	/	KF824598	/
			*G. monticellus*	/	/	KF824607	/
			*G. benignus*	/	/	KF824589	/
			*G. incoercitus*	/	/	KF824588	/

Note

The bold font indicate species that more evidence is needed to define those species; the underlined font indicates reference sequence.

In view of the differences between the morphological and molecular identification results, when calculating the genetic distance, *N. davidi davidi*, *N. davidi koreana,* and *P. modestus*, *M*. sp. “*qilianensis*” were considered as separate species. At the same time, we combined *N. davidi davidi* and *N. davidi koreana* as *Neocaridina davidi*, *P. modestus,* and *M*. sp. “*qilianensis*” as *Palaemon modestus* (in the following these are expressed by the full names, *Neocaridina davidi* and *Palaemon modestus*) for the estimation of the paired genetic distances according to the results of morphological and phylogenetic tree analyses. Mean K2P distances and p‐distances within species were both equal to 1.2%. The maximum K2P distances of all species were less than 2%, with the exceptions of *Palaemon modestus* (2.5%) and *M. maculatum* (2.3%). Similarly, the maximum p‐distances of all species were also less than 2%, with the exceptions of *Palaemon modestus* (2.5%) and *M. maculatum* (2.2%). The results showed that both the K2P and the p‐distances produced similar results in genetic distance and phylogenetic analysis. Furthermore, relatively high genetic divergence was also detected in *M. maculatum, Neocaridina davidi,* and *P. sinensis* (Table 2).

**TABLE 2:** The genetic distances of the four‐water system freshwater shrimp populations in Henan Province

Species	Group	K2P	1	2	3	4	5	6	7	8	9	10	11	12	13	14	15
*Macrobrachium maculatum*	1	**0.023**	**0.022**	0.251	0.188	0.161	0.199	0.219	0.244	0.203	0.196	0.203	0.193	0.192	0.188	0.190	0.198
*Gammarus pisinnus*	2	**0.003**	0.308	**0.003**	0.265	0.269	0.259	0.291	0.224	0.261	0.262	0.257	0.267	0.267	0.275	0.266	0.259
*M*. sp. “*qilianensis*”	3	**0.005**	0.217	0.330	**0.005**	0.181	0.205	0.223	0.229	0.198	0.203	0.209	0.198	0.042	0.190		0.204
*Macrobrachium nipponense*	4	**0.013**	0.184	0.337	0.209	**0.012**	0.194	0.228	0.240	0.191	0.196	0.205	0.189	0.193	0.187	0.187	0.194
*N. davidi koreana*	5	**0.011**	0.234	0.320	0.241	0.227	**0.011**	0.216	0.247	0.047	0.024	0.072	0.061	0.212	0.195	0.209	
*Caridina gracilipes*	6	**0.001**	0.263	0.372	0.267	0.275	0.260	**0.001**	0.263	0.222	0.217	0.218	0.218	0.235	0.232	0.230	0.216
*Procambarus clarkii*	7	**0.006**	0.299	0.268	0.278	0.293	0.304	0.329	**0.006**	0.236	0.249	0.235	0.243	0.229	0.262	0.229	0.248
*Neocaridina denticulata denticulata*	8	**0.006**	0.239	0.323	0.231	0.224	0.049	0.269	0.287	**0.006**	0.043	0.074	0.056	0.206	0.194	0.203	0.046
*N. davidi davidi*	9	**0.001**	0.230	0.326	0.238	0.231	0.025	0.262	0.307	0.045	**0.001**	0.070	0.051	0.209	0.188	0.206	
*Neocaridina ikiensis*	10	**n/c**	0.240	0.318	0.247	0.243	0.076	0.263	0.284	0.079	0.074	**n/c**	0.081	0.210	0.190	0.210	0.071
*Neocaridina palmata*	11	**n/c**	0.226	0.334	0.231	0.220	0.065	0.262	0.298	0.059	0.053	0.086	**n/c**	0.210	0.196	0.205	0.059
*P. modestus*	12	**0.009**	0.223	0.334	0.043	0.226	0.251	0.286	0.277	0.242	0.247	0.249	0.248	**0.008**	0.200		0.211
*Palaemon sinensis*	13	**0.016**	0.219	0.346	0.222	0.218	0.227	0.282	0.329	0.227	0.218	0.220	0.229	0.236	**0.016**	0.195	0.193
***Palaemon modestus***	14	**0.025**	0.220	0.332		0.218	0.247	0.277	0.278	0.237	0.243	0.248	0.240		0.23	**0.025**	0.208
***Neocaridina davidi***	15	**0.016**	0.198	0.259	0.259	0.194		0.216	0.248	0.046		0.071	0.059	0.211	0.193	0.246	**0.016**

Note

The list of K2P is K2P genetic distance within populations; diagonal bold is P‐distance genetic distances within populations; below diagonal is K2P genetic distance among populations; above diagonal is P‐distance genetic distance among populations.

The results of the ABGD analysis showed that when the value of the prior intraspecific divergence was 0.035938, the recursive partition and initial partition tended to be the same. In the ABGD analysis, the freshwater shrimp were divided into eight MOTUs; the division results are shown in Figure 2. *N. ikiensis* (M0301), *N. palmata* (S0400), *N. davidi koreana,* and *N. davidi davidi* were identified as one species. Meanwhile, *M*. sp. “*qilianensis*” and *P. modestus* were identified as one species.

We uploaded the haplotype ML tree of 222 *COI* freshwater shrimp sequences to https://species.h‐its.org/ptp, set the tree as unrooted, set the number of MCMC generations to 100,000, and other settings as the default parameters. The results of the division were as follows: the estimated species number of the 222 *COI* sequences was between 13 and 15 based on bPTP analysis; maximum likelihood (ML) divided the 222 *COI* sequences into 13 MOTUs, but the highest supported solution of the Bayesian inference (BI) divided the sequences into 15 MOTUs. The results showed that the estimated species number of bPTP (BI) was much larger than the number of species classified by morphology; *Caridina gracilipes* and *Palaemon sinensis* were divided into two MOTUs, and there was obvious over‐classification. We selected the definition results of the bPTP (ML) analysis (Figure 2). Consistent with the results of the NJ phylogenetic tree, *M*. sp. “*qilianensis*” and *P. modestus* and *N. davidi davidi* and *N. davidi koreana* were divided into sister clades and independent MOTUs in the bPTP analysis. At the same time, *N. ikiensis* (M0301) and *N. palmata* (S0400) were identified as independent MOTUs.

## 4 DISCUSSION

### 4.1 Barcoding success

It is well known that taxonomic identification of organisms is the most fundamental and important task of all biological research (Luo et al., 2018). The early classification identification was mainly based on detailed morphological characteristics observation and anatomical structure verification by professional taxonomists; however, this task needs significant time and has high requirements for researchers and experimental specimens (Carvalho et al., 2011; Hebert, Ratnasingham, et al., 2003; Shen et al., 2016). In addition, there is always the demise of existing species and the emergence of new species; with the rapid development of science and technology, increasing numbers of new species have been discovered, so that the number of specialists in alpha taxonomy is not sufficient to carry out extensive and complex morphological identification (Oliver, 2015). Our traditional morphological identification results showed that there are nine species of freshwater shrimp in Henan Province. On the whole, there was more obvious morphological variation in the widespread taxa such as *Macrobrachium*, *Palaemon,* and *Neocaridina*. The rostrum variation in shape, length, and number of serrations of some individuals of *N. denticulate*, *Neocaridina davidi*, *M. maculatum,* and *M. nipponense* was obvious, even exceeding the definition range of those species’ descriptions, and this may be caused by their wide distributions and geographical separation (Li et al., 2007; Liang, 2004). In addition, due to the severe morphological damage, samples S0400 and M0301 could not be identified. Therefore, traditional taxonomic recognition is not only complicated and difficult, but also not conducive to widespread implementation.

With the development of modern technology and the arrival of the molecular era, molecular identification has gradually become popular and has been widely used in biological identification. Since the first use of *COI* for species identification, it has been shown that this gene fragment can be used in “DNA barcoding” for biological authentication in many invertebrate species (Barrett & Hebert, 2005; Clare et al., 2007; Hebert, Ratnasingham, et al., 2003; Hendrich et al., 2014). The research of Costa and Mar and colleagues further demonstrated that barcode technology is efficient and accurate in the species identification of the freshwater shrimp (Costa et al., 2007; Mar et al., 2018). Our study showed that both the identification results of the NJ phylogenetic analysis and the bPTP analysis identified at least 13 MOTUs among the freshwater shrimp in Henan Province. There was a close evolutionary relationship between *M*. sp. “*qilianensis*” and *P. modestus*, *N. davidi koreana,* and *N. davidi davidi*; they are sister clades. The ABGD analysis identified eight species, among which *N. ikiensis* (M0301), *N. palmata* (S0400), and two subspecies of *Neocaridina davidi* were identified as one species. Meanwhile, *M*. sp. “*qilianensis*” and *P. modestus* were also identified as the same species. According to the NJ tree, bPTP analysis and ABGD analysis estimated the paired genetic distances of freshwater prawns in Henan Province. When the 13 MOTUs were treated as single taxa, the intraspecific genetic distances of the other taxa were all less than 0.02, except for *M. maculatum* (0.023). When combining *N. davidi koreana* with *N. davidi davidi* as a taxon, the genetic distance was 0.016. However, when *M*. sp. “*qilianensis*” and *P. modestus* were calculated as a whole, the genetic distance within species was 0.025, beyond the intraspecific threshold. Our molecular identification results show that *COI* DNA barcode technology can not only effectively identify species identified by morphology but also identify species that are nearly identical in terms of morphology.

The results of the study show that all nine species identified by traditional morphology could be further divided and confirmed by molecular methods. The molecular analysis identified *N. ikiensis* (M0301), *N. palmata* (S0400), and *M*. sp. “*qilianensis*,” three additional species. *N. ikiensis* (M0301) and *N. palmata* (S0400) were morphologically identified as *Neocaridina* due to severe morphological damage. Our study has shown that the number of species identified by molecular biological identification is usually higher than that using traditional morphology, and it also demonstrated that the *COI* DNA barcode technology is efficient in the species identification of freshwater shrimp.

### 4.2 Species diversity

The morphological identification results showed that there are nine species of freshwater shrimp in Henan Province. Compared with the study of Wang (1989), our sampling points covered his 15 sampling points plus the main river systems and tributaries in Henan Province. Unfortunately, we have not collected and identified *Macrobrachium superbum*, *Macrobrachium asperulum*, or *Macrobrachium iusulare*. In order to avoid the single sampling error, we repeatedly went to the collection sites where the distributions were recorded, and the collection range was further expanded. Even so, we have not collected these species. The records indicate that the above three freshwater shrimp are mainly distributed in some provinces and waters of southern China (Li et al., 2007), and the morphological characteristics of *Macrobrachium* are similar, making the species difficult to identify. Therefore, we hypothesize that these species may have existed in Henan Province before, but the environmental changes of the sample sites may have proven unsuitable for these species and that they have migrated or disappeared from the province. In addition, they may never have been distributed in Henan Province, and similar morphological characteristics may have led to their incorrect identification. All in all, more samples and more direct evidence are needed to support the existence of these species in Henan Province.

At present, the classification status of a variety of freshwater shrimp has changed, indirectly hindering the effective identification of their species and the estimation of biodiversity. First, the taxonomic status of *Caridina denticulata sinensis* (Kemp, 1918) and *Palaemon* (*Exopalaemon*) *modestus* (Heller, 1862) collected by Wang has been controversial and has changed to some extent (Wang, 1989). As early as 1918, Kemp regarded the *Caridina* specimens collected from Taihu Lake as a new subspecies of *Caridina denticulata* and named it *C. denticulata sinensis*. Kubo separated *C. denticulata* from *Caridina* to form the genus *Neocaridina* in 1938. Due to the small number of species and this being based on morphological traits, the name *Neocaridina* has not been widely adopted. Cai confirmed the taxonomic status of the genus *Neocaridina* in 1996 and revised it (Cai, 1996). In this revision, Cai considered that *C. davidi* (Bouvier, 1904) was a subspecies of *N. denticulata* (*N. denticulata davidi*) and transferred it to the genus *Neocaridina*. However, Liang considered *C. davidi* (Bouvier, 1904), *N. denticula davidi* (Kubo, 1938), and *N. denticula sinensis* (Kemp, 1913) as synonyms of *Neocaridina heteropoda heteropoda* (Liang, 2002). Our molecular and morphological identification results also confirmed this point (Klotz et al., 2013; Liang, 2004). Klotz pointed out that *N. denticulata sinensis* reported by Englund and Cai (1999) and *N. davidi* reported here are conspecifics (Klotz et al., 2013). Here, we followed Klotz et al. (2013) and considered that *C. davidi* (Bouvier, 1904) as the senior synonym has clear priority (article 23 of the ICZN), and we continue to name it *N. davidi* (Klotz et al., 2013). In addition, *Palaemonetes*, *Exopalaemon*, and *Coutierella* have been transferred to *Palaemon,* and this is widely accepted (Ashelby et al., 2012). Due to the genus classification status changes, *Palaemon* *(Exopalaemon)* *modestus* should be renamed *Palaemon modestus*, and *Palaemonetes sinensis* should also be renamed as *Palaemon sinensis*.

Second, due to the failure to identify enough morphological differentiation in *M*. sp. “*qilianensis*,” and the lack of a sufficient description in the relevant references and original literature, we tentatively inferred that *M*. sp. “*qilianensis*” may be an invalid species and that it may be a synonym of *Palaemon modestus*. In addition, given that only one sample was obtained, and *N. ikiensis* and *N. palmata* were damaged, they cannot be effectively identified by morphology. Thus, *N. ikiensis* and *N. palmata* need to be further collected and confirmed.

In conclusion, the comprehensive results of morphological characteristics and molecular delimitation indicated that there are at least nine species of freshwater shrimp that have been morphologically identified in Henan Province.

### 4.3 Cryptic species

The aims of DNA barcoding are identification of unknown specimens via DNA barcodes of a priori defined taxonomic entities in databases (Merckelbach & Borges, 2020). The method is being increasingly utilized to tackle many issues, including illegal species exploitation, food fraud, biological invasions, and biodiversity monitoring (Bohmann et al., 2014; Gonçalves et al., 2015; Hubert et al., 2015; Khaksar et al., 2015). The DNA barcode solves the problem of molecular delimitation of species to a certain extent, but to rely on it exclusively is far from sufficient to solve the delimitation of species and the discovery of cryptic species. In this study, a variety of DNA barcode analyses were used to identify freshwater shrimp species. The NJ tree, genetic distance, and PTP analyses indicated that the genetic differentiation of *Neocaridina davidi, M. maculatum*, *M. nipponense,* and *Palaemon modestus* was clear; all had formed no less than one separate cluster or monophyletic clade. The intraspecific genetic distances of *M. maculatum* and *Palaemon modestus* were more than 2%. Both molecular and morphological characteristics showed that there were significant genetic differentiation and morphological differences between the above species, but there is no definitive criterion for whether these differences are sufficient to indicate the emergence of a new species or the existence of an underlying species.

In the process of speciation, the boundaries of new species become clearer over time. However, before the completion of this process (known as gray zone sense), the boundaries between species are often fuzzy and difficult to recognize. Cryptic species are the intermediate products or even final products of this process (De Queiroz, 2007). Species delimitation studies are dedicated to defining the species that are unknown or problematic by compiling molecular, morphological, and karyotype data (Kekkonen & Hebert, 2014). This analysis is usually applicable to the groups for which there has been substantial research, but its ability to define many taxonomic species with less basic knowledge and description is limited (Common, 1990; Raven & Yeates, 2014). In fact, even though there is sufficient evidence to support the species hypothesis and species delimiting, there are still many newly discovered species that have not been described (Pante et al., 2015), a situation that hinders taxonomic progress, species identification, and biodiversity estimation (Schlick‐Steiner et al., 2007). Thus, if a species is marked as merely presumed rather than formally described and therefore fully established, the taxonomy is still incomplete; so, the transition from species delimitation to species description is still a major task to be accomplished (Merckelbach & Borges, 2020; Miralles & Vences, 2013).

In our results, the delimitation of almost all species of freshwater shrimp was in accordance with the genetic and morphological definitions, and most of the molecular delimitation analyses showed a higher species number than those indicated by morphological identification. This suggests that there are likely to be cryptic species that have yet to be identified and described, even if they are not sufficiently differentiated to support the formation of a single new species. The analysis also shows that the ability of DNA barcodes to identify the undescribed species from recent speciation events is limited, although it can be widely used to identify new taxa in complex groups, identify unknown species, and find cryptic species (Iyiola et al., 2018). Further studies and descriptions of species are needed to determine whether the intermediate process of a species’ differentiation is sufficient to form a new species, and whether there are cryptic species.

## CONFLICT OF INTERESTS

All authors declare that they have no competing interests.

## AUTHOR CONTRIBUTIONS

**Chuan‐jiang Zhou:** Conceptualization (lead); Funding acquisition (lead); Investigation (lead); Supervision (lead); Writing‐review & editing (lead). **Meng‐xia Feng:** Data curation (lead); Investigation (supporting); Methodology (lead); Software (lead); Writing‐original draft (lead). **Yong‐tao Tang:** Investigation (supporting); Writing‐review & editing (supporting). **Chang‐xing Yang:** Investigation (supporting). **Xiao‐lin Meng:** Investigation (supporting). **Guo‐xing Nie:** Conceptualization (lead); Funding acquisition (lead); Investigation (lead); Supervision (lead).

## Supporting information

Figure S1

Table S1

Table S2

## Data Availability

DNA sequences have been deposited in GenBank under Accession numbers MW069488–MW069709. Details regarding individual samples are available in Table S2.

## References

[ece37855-bib-0001] Ashelby, C. W. , Page, T. J. , De Grave, S. , Hughes, J. M. , & Johnson, M. L. (2012). Regional scale speciation reveals multiple invasions of freshwater in Palaemoninae (Decapoda). Zoologica Scripta, 41, 293–306. 10.1111/j.1463-6409.2012.00535.x

[ece37855-bib-0002] Barrett, R. D. H. , & Hebert, P. D. N. (2005). Identifying spiders through DNA barcodes. Canadian Journal of Zoology, 83, 481–491. 10.1139/z05-024

[ece37855-bib-0003] Birch, J. L. , Walsh, N. G. , Cantrill, D. J. , Holmes, G. D. , & Murphy, D. J. (2017). Testing efficacy of distance and tree‐based methods for DNA barcoding of grasses (Poaceae tribe *Poeae*) in Australia. PLoS One, 12(10), e0186259. 29084279 10.1371/journal.pone.0186259 PMC5662090

[ece37855-bib-0004] Blair, C. , & Bryson, R. W. (2017). Cryptic diversity and discordance in single‐locus species delimitation methods within horned lizards (Phrynosomatidae: *Phrynosoma*). Molecular Ecology Resources, 17, 1168–1182. 28161911 10.1111/1755-0998.12658

[ece37855-bib-0005] Bohmann, K. , Evans, A. , Gilbert, M. T. P. , Carvalho, G. R. , Creer, S. , Knapp, M. , Yu, D. W. , & de Bruyn, M. (2014). Environmental DNA for wildlife biology and biodiversity monitoring. Trends in Ecology & Evolution, 29, 358–367. 10.1016/j.tree.2014.04.003 24821515

[ece37855-bib-0006] Burghart, S. E. , Woudenberg, L. V. , Daniels, C. A. , Meyers, S. D. , Peebles, E. B. , & Breitbart, M. (2014). Disparity between planktonic fish egg and larval communities as indicated by DNA barcoding. Marine Ecology Progress Series, 503, 195–204. 10.3354/meps10752

[ece37855-bib-0007] Cai, Y. (1996). A revision of the genus *Neocaridina* (Crustacea: Decapoda: Atyidae). Acta Zootaxonomica Sinica, 21, 129–160.

[ece37855-bib-0008] Cai, Y. X. , & Ng, P. K. L. (2002). The freshwater palaemonid prawns (Crustacea: Decapoda: Caridea) of Myanmar. Hydrobiologia, 487, 59–83.

[ece37855-bib-0009] Carvalho, D. C. , Neto, D. A. P. , Brasil, B. S. A. F. , & Oliveira, D. A. A. (2011). DNA barcoding unveils a high rate of mislabeling in a commercial freshwater catfish from Brazil. Mitochondrial DNA, 22, 97–105. 10.3109/19401736.2011.588219 21707317

[ece37855-bib-0555] Cheng, Q. Q. (2009). https://www.ncbi.nlm.nih.gov/nuccore/FJ958200

[ece37855-bib-0010] Chang, C. H. , Lin, H. Y. , Ren, Q. , Lin, Y. S. , & Shao, K. T. (2016). DNA barcode identification of fish products in Taiwan: Government‐commissioned authentication cases. Food Control, 66, 38–43. 10.1016/j.foodcont.2016.01.034

[ece37855-bib-0011] Clare, E. L. , Lim, B. K. , Engstrom, M. D. , Eger, J. L. , & Ebert, P. D. N. (2007). DNA barcoding of Neotropical bats: Species identification and discovery within Guyana. Molecular Ecology Notes, 7, 184–190. 10.1111/j.1471-8286.2006.01657.x

[ece37855-bib-0012] Collins, R. A. , Boykin, L. M. , Cruickshank, R. H. , & Armstrong, K. F. (2012). Barcoding's next top model: An evaluation of nucleotide substitution models for specimen identification. Methods in Ecology and Evolution, 3, 457–465. 10.1111/j.2041-210X.2011.00176.x

[ece37855-bib-0013] Common, I. F. B. (1990). Moths of Australia. Melbourne University Press.

[ece37855-bib-0014] Costa, F. O. , deWaard, J. R. , Boutillier, J. , Ratnasingham, S. , Dooh, R. T. , Hajibabaei, M. , & Hebert, P. D. N. (2007). Biological identifications through DNA barcodes: The case of the Crustacea. Canadian Journal of Fisheries and Aquatic Science, 64, 272–295. 10.1139/f07-008

[ece37855-bib-0015] De Grave, S. , Cai, Y. X. , & Anker, A. (2008). Global diversity of shrimps (Crustacea: Decapoda: Caridea) in freshwater. Hydrobiologia, 595, 287–293. 10.1007/s10750-007-9024-2

[ece37855-bib-0016] De Grave, S. , Li, C. P. , Tsang, L. M. , Chu, K. H. , & Chan, T. Y. (2014). Unweaving hippolytoid systematics (Crustacea, Decapoda, Hippolytidae): Resurrection of several families. Zoologica Scripta, 43, 496–507. 10.1111/zsc.12067

[ece37855-bib-0017] De Grave, S. , Smith, K. G. , Adeler, N. A. , Allen, D. J. , Alvarez, F. , Anker, A. , Cai, Y. , Carrizo, S. F. , Klotz, W. , Mantelatto, F. L. , Page, T. J. , Shy, J.‐Y. , Villalobos, J. L. , & Wowor, D. (2015). Dead shrimp blues: A global assessment of extinction risk in freshwater shrimps (Crustacea: Decapoda: Caridea). PLoS One, 10, 1–14. 10.1371/journal.pone.0120198 PMC4373683 25807292

[ece37855-bib-0018] Deng, Y. , & Wu, B. (1997). A preliminary investigating on the shrimp in Guizhou. Chinese Journal of Zoology, 032, 5–8.

[ece37855-bib-0019] DeSalle R. , Egan M. G , Siddall M. (2005). The unholy trinity: taxonomy, species delimitation and DNA barcoding. Philosophical Transactions of the Royal Society B: Biological Sciences, 360, (1462), 1905–1916. 10.1098/rstb.2005.1722 PMC1609226 16214748

[ece37855-bib-0020] Englund, R. A. , & Cai, Y. X. (1999). The occurrence and description of *Neocaridina denticulata sinensis* (Kemp, 1918) (Crustacea: Decapoda: Atyidae), a new introduction to the Hawaiian Islands. Bishop Museum Occasional Papers, 58, 58–65.

[ece37855-bib-0021] Feng, J. , Sun, Y. , Cheng, X. , & Li, J. (2008). Sequence analysis of mitochondrial *COI* gene of *Macrobrachium nipponense* from the five largest freshwater lakes in China. Journal of Fisheries, 4, 23–31.

[ece37855-bib-0022] Folmer, O. , Black, M. B. , Wr, H. , Lutz, R. , & Vrijenhoek, R. (1994). DNA primers for amplification of mitochondrial Cytochrome *c* oxidase subunit I from diverse metazoan invertebrates. Molecular Marine Biology and Biotechnology, 3, 294–299. 7881515

[ece37855-bib-0023] Gonçalves, P. F. M. , Oliveira‐Marques, A. R. , Matsumoto, T. E. , & Miyaki, C. Y. (2015). DNA barcoding identifies illegal parrot trade. Journal of Heredity, 106, 560–564. 10.1093/jhered/esv035 26245790

[ece37855-bib-0024] Hebert, P. D. N. , Cywinska, A. , Ball, S. L. , & deWaard, J. R. (2003). Biological identifications through DNA barcodes. Proceedings of the Royal Society of London. Series B: Biological Sciences, 270, 313–321. 10.1098/rspb.2002.2218 12614582 PMC1691236

[ece37855-bib-0025] Hebert, P. D. N. , & Gregory, T. R. (2005). The promise of DNA barcoding for taxonomy. Systematic Biology, 54, 852–859. 10.1080/10635150500354886 16243770

[ece37855-bib-0026] Hebert, P. D. N. , Ratnasingham, S. , & de Waard, J. R. (2003). Barcoding animal life: Cytochrome c oxidase subunit 1 divergences among closely related species. Proceedings of the Royal Society of London. Series B: Biological Sciences, 270, S96–S99. 12952648 10.1098/rsbl.2003.0025 PMC1698023

[ece37855-bib-0027] Hendrich, L. , Morinière, J. , Haszprunar, G. , Hebert, P. D. N. , Hausmann, A. , Köhler, F. , & Balke, M. (2014). A comprehensive DNA barcode database for Central European beetles with a focus on Germany: Adding more than 3500 identified species to BOLD. Molecular Ecology Resources, 15, 795–818. 25469559 10.1111/1755-0998.12354

[ece37855-bib-0028] Holthuis, L. B. (1980). Shrimps and prawns of the world. Food and Agriculture Organization of the United Nations.

[ece37855-bib-0029] Hubert, N. , Espiau, B. , Meyer, C. , & Planes, S. (2015). Identifying the ichthyoplankton of a coral reef using DNA barcodes. Molecular Ecology Resources, 15, 57–67. 10.1111/1755-0998.12293 24935524

[ece37855-bib-0030] Hunte, W. (1978). The distribution of freshwater shrimps (Atyidae and Palaemonidae) in Jamaica. Zoological Journal of the Linnean Society, 64, 135–150. 10.1111/j.1096-3642.1978.tb01065.x

[ece37855-bib-0031] Iyiola, O. A. , Nneji, L. M. , Mustapha, M. K. , Nzeh, C. G. , Oladipo, S. O. , Nneji, I. C. , Okeyoyin, A. O. , Nwani, C. D. , Ugwumba, O. A. , Ugwumba, A. A. A. , Faturoti, E. O. , Wang, Y.‐Y. , Chen, J. , Wang, W.‐Z. , & Adeola, A. C. (2018). DNA barcoding of economically important freshwater fish species from north‐central Nigeria uncovers cryptic diversity. Ecology and Evolution, 8, 6932–6951. 10.1002/ece3.4210 30073057 PMC6065348

[ece37855-bib-0033] Kekkonen, M. , & Hebert, P. D. N. (2014). DNA barcode‐based delineation of putative species: Efficient start for taxonomic workflows. Molecular Ecology Resources, 14, 706–715. 24479435 10.1111/1755-0998.12233 PMC4264940

[ece37855-bib-0034] Khaksar, R. , Carlson, T. , Schaffner, D. W. , Ghorashi, M. , Best, D. , Jandhyala, S. , Traverso, J. , & Amini, S. (2015). Unmasking seafood mislabeling in U.S. markets: DNA barcoding as a unique technology for food authentication and quality control. Food Control, 56, 71–76.

[ece37855-bib-0035] Klotz, W. , Miesen, F. W. , Hüllen, S. , & Herder, F. (2013). Two Asian fresh water shrimp species found in a thermally polluted stream system in North Rhine‐Westphalia, Germany. Aquatic Invasions, 8, 333–339. 10.3391/ai.2013.8.3.09

[ece37855-bib-0036] Kumar, S. , Stecher, G. , & Tamura, K. (2016). MEGA7: Molecular evolutionary genetics analysis Version 7.0 for Bigger Datasets. Molecular Biology and Evolution, 33, 1870–1874. 10.1093/molbev/msw054 27004904 PMC8210823

[ece37855-bib-0037] Lee, S. J. , & Kim, J. K. (2014). Identification of *Trichiurus* (Pisces: Trichiuridae) eggs and larvae from Korea, with a Taxonomic Note. Fisheries and Aquatic Sciences, 17, 137–143. 10.5657/FAS.2014.0137

[ece37855-bib-0038] Li, X. , Liu, R. , Liang, X. , & Chen, G. (2007). Fauna Sinica. Invertebrata, vol. 44. Crustacea Decapoda Palaemonoidea. Science Press.

[ece37855-bib-0039] Liang, X. (2004). Fauna Sinica. Invertebrata, vol. 36. Crustacea. Decapoda. Atyidae. Science Press.

[ece37855-bib-0040] Librado, P. , & Rozas, J. (2009). DnaSP v5: A software for comprehensive analysis of DNA polymorphism data. Bioinformatics, 25, 1451–1452. 10.1093/bioinformatics/btp187 19346325

[ece37855-bib-0041] Liu, R. (1955). Economic shrimp in northern China. Science Press.

[ece37855-bib-0042] Luo, A. R. , Cheng, L. , Ho, S. Y. W. , & Zhu, C. D. (2018). Comparison of methods for molecular species delimitation across a range of speciation scenarios. Systematic Biology, 67, 830–846. 10.1093/sysbio/syy011 29462495 PMC6101526

[ece37855-bib-0043] Macher, J. N. , Macher, T. H. , & Leese, F. (2017). Combining NCBI and BOLD databases for OTU assignment in metabarcoding and metagenomic datasets: The BOLD_NCBI_Merger. PeerJ, 1, e22262. 10.3897/mbmg.1.22262

[ece37855-bib-0044] Makombu, J. G. , Stomeo, F. , Oben, P. M. , Tilly, E. , Stephen, O. O. , Mujibi, D. N. , Cheruiyot, E. K. , Tarekegn, G. M. , Zango, P. , Egbe, A. E. , Ndagyong, A. , Mialhe, E. , & Ngueguim, J. R. (2019). Morphological and molecular characterization of freshwater prawn of genus *Macrobrachium* in the coastal area of Cameroon. Ecology and Evolution, 9, 14217–14233. 31938513 10.1002/ece3.5854 PMC6953584

[ece37855-bib-0045] Mar, W. , Kang, P. F. , Mao, B. , & Wang, Y. F. (2018). Morphological and molecular features of some freshwater prawn species under genus *Macrobrachium* Spence Bate, 1868 (Crustacea: Decapoda: Palaemonidae) from Myanmar. Zootaxa, 4388, 123–132. 10.11646/zootaxa.4388.1.9 29690469

[ece37855-bib-0046] Martin, J. W. , & Davis, G. E. (2001). An updated classification of the recent crustacea. Los Angeles: Natural History Museum of Los Angeles County, Science Series, 39, 1–124.

[ece37855-bib-0047] Merckelbach, L. M. , & Borges, L. M. S. (2020). Make every species count: FastaChar software for rapid determination of molecular diagnostic characters to describe species. Molecular Ecology Resources, 20, 1761–1768. 32623815 10.1111/1755-0998.13222

[ece37855-bib-0048] Miralles, A. , & Vences, M. (2013). New metrics for comparison of taxonomies reveal striking discrepancies among species delimitation methods in *Madascincus* lizards. PLoS One, 8, e68242. 10.1371/journal.pone.0068242 23874561 PMC3710018

[ece37855-bib-0049] New, M. B. , & Nair, C. M. (2012). Global scale of freshwater prawn farming. Aquaculture Research, 43, 960–969. 10.1111/j.1365-2109.2011.03008.x

[ece37855-bib-0050] Oliver, C. C. (2015). Taxonomy in times of the taxonomic impediment – examples from the community of experts on amphipod crustaceans. Journal of Crustacean Biology, 35, 729–740. 10.1163/1937240X-00002381

[ece37855-bib-0051] Pante, E. , Schoelinck, C. , & Puillandre, N. (2015). From integrative taxonomy to species description: One step beyond. Systematic Biology, 64(1), 152–160. 10.1093/sysbio/syu083 25358968

[ece37855-bib-0052] Pont, D. , Rocle, M. , Valentini, A. , Civade, R. , Jean, P. , Maire, A. , Roset, N. , Schabuss, M. , Zornig, H. , & Dejean, T. (2018). Environmental DNA reveals quantitative patterns of fish biodiversity in large rivers despite its downstream transportation. Scientific Reports, 8, 10361–10373. 10.1038/s41598-018-28424-8 29991759 PMC6039509

[ece37855-bib-0053] Puillandre, N. , Lambert, A. , Brouillet, S. , & Achaz, G. (2012). ABGD, Automatic Barcode Gap Discovery for primary species delimitation. Molecular Ecology, 21, 1864–1877. 10.1111/j.1365-294X.2011.05239.x 21883587

[ece37855-bib-0054] Queiroz, K. D. (2007). Species concepts and species delimitation. Systematic Biology, 56, 879–886. 10.1080/10635150701701083 18027281

[ece37855-bib-0055] Raven, P. H. , & Yeates, D. K. (2014). Australian biodiversity: Threats for the present, opportunities for the future. Austral Entomology, 46, 177–187. 10.1111/j.1440-6055.2007.00601.x

[ece37855-bib-0056] Sambrook, J. , & Russel, D. W. (2001). Molecular Cloning: A laboratory manual (3rd ed.). Cold Spring Harbor Laboratory.

[ece37855-bib-0057] Schlick‐Steiner, B. C. , Seifert, B. , Stauffer, C. , Christian, E. , Crozier, R. H. , & Steiner, F. M. (2007). Without morphology, cryptic species stay in taxonomic crypsis following discovery. Trends in Ecology & Evolution, 22, 391–392. 10.1016/j.tree.2007.05.004 17573150

[ece37855-bib-0058] Schlick‐Steiner, B. C. , Steiner, F. M. , Seifert, B. , Stauffer, C. , Christian, E. , & Crozier, R. H. (2010). Integrative Taxonomy: A multisource approach to exploring biodiversity. Annual Review of Entomology, 55, 421–438. 10.1146/annurev-ento-112408-085432 19737081

[ece37855-bib-0059] Shen, Y. J. , Guan, L. H. , Wang, D. Q. , & Gan, X. N. (2016). DNA barcoding and evaluation of genetic diversity in Cyprinidae fish in the midstream of the Yangtze River. Ecology and Evolution, 6, 2702–2713. 27066250 10.1002/ece3.2060 PMC4798831

[ece37855-bib-0060] Srivathsan, A. , & Meier, R. (2012). On the inappropriate use of Kimura‐2‐parameter (K2P) divergences in the DNA‐barcoding literature. Cladistics, 28, 190–194. 10.1111/j.1096-0031.2011.00370.x 34861755

[ece37855-bib-0061] Stamatakis, A. (2014). RAxML version 8: A tool for phylogenetic analysis and post‐analysis of large phylogenies. Bioinformatics, 30, 1312–1313. 10.1093/bioinformatics/btu033 24451623 PMC3998144

[ece37855-bib-0062] Suzuki, H. , Tanigawa, N. , Nagatomo, T. , & Tsuda, E. (1993). Distribution of freshwater caridean shrimps and prawns (Atyidae and Palaemonidae) from Southern Kyushu and adjacent islands, Kagoshima Prefecture, Japan. Crustacean Research, 22, 55–64. 10.18353/crustacea.22.0_55

[ece37855-bib-0063] Swindell, S. R. , & Plasterer, T. N. (1997). SEQMAN. Contig Assembly. Methods in Molecular Biology, 70, 75–89. 9089604

[ece37855-bib-0064] Takahara, T. , Minamoto, T. , & Doi, H. (2013). Using environmental DNA to estimate the distribution of an invasive fish species in ponds. PLoS One, 8, e56584. 10.1371/journal.pone.0056584 23437177 PMC3577852

[ece37855-bib-0065] Tinacci, L. , Guidi, A. , Toto, A. , Guardone, L. , Giusti, A. , D'Amico, P. , & Armani, A. (2018). DNA barcoding for the verification of supplier's compliance in the seafood chain: How the lab can support companies in ensuring traceability. Italian Journal of Food Safety, 7, 6894–6899. 10.4081/ijfs.2018.6894 30046552 PMC6037000

[ece37855-bib-0066] Tippmann, H. (2004). Software review: Analysis for free: Comparing programs for sequence analysis. Briefings in Bioinformatics, 5, 82–87. 10.1093/bib/5.1.82 15153308

[ece37855-bib-0067] Wang, F. (1989). A preliminary study on shrimp from Henan Province. Henan Fisheries, 16–18.

[ece37855-bib-0068] Wang, Z. , Guo, Y. , Chen, R. , He, X. , Liu, C. , & Liu, Y. (2009). *COI* barcode sequences of teleosts in the South China Sea. Oceanologia et Limnologia Sinica, 40, 608–614.

[ece37855-bib-0069] Wong, E.‐H.‐K. , & Hanner, R. H. (2008). DNA barcoding detects market substitution in North American seafood. Food Research International, 41, 828–837. 10.1016/j.foodres.2008.07.005

[ece37855-bib-0070] Wong, L. L. , Peatman, E. , Lu, J. , Kucuktas, H. , He, S. , Zhou, C. , Na‐nakorn, U. , & Liu, Z. (2011). DNA barcoding of catfish: Species authentication and phylogenetic assessment. PLoS One, 6, e17812. 10.1371/journal.pone.0017812 21423623 PMC3057997

[ece37855-bib-0071] Zhang, J. J. , Kapli, P. , Pavlidis, P. , & Stamatakis, A. (2013). A general species delimitation method with applications to phylogenetic placements. Bioinformatics, 29, 2869–2876. 10.1093/bioinformatics/btt499 23990417 PMC3810850

[ece37855-bib-0072] Zhang, Q. , Cheng, Q. , & Guan, W. (2009). Mitochondrial *COI* gene sequence variation and taxonomic status of three *Macrobrachium* species. Zoological Research, 30, 613–619.

[ece37855-bib-0073] Zheng, M. (1989). Investigation of freshwater shrimps in Jianxi water system, Fujian. Chinese Journal of Zoology, 24(6), 7–11.

[ece37855-bib-0074] Zhu, Q. , & Miao, Y. (1990). Jiangsu freshwater shrimp and its fishery. Chinese Journal of Zoology, 25, 8–11.

