# Supplementary figures and images for: Species diversity of freshwater shrimp in Henan Province, China, based on morphological characters and COI mitochondrial gene
Source: Ecol Evol. 2021 Jul 13;11(15):10502–14. doi: 10.1002/ece3.7855 (PMC8328406; doi:10.1002/ece3.7855)

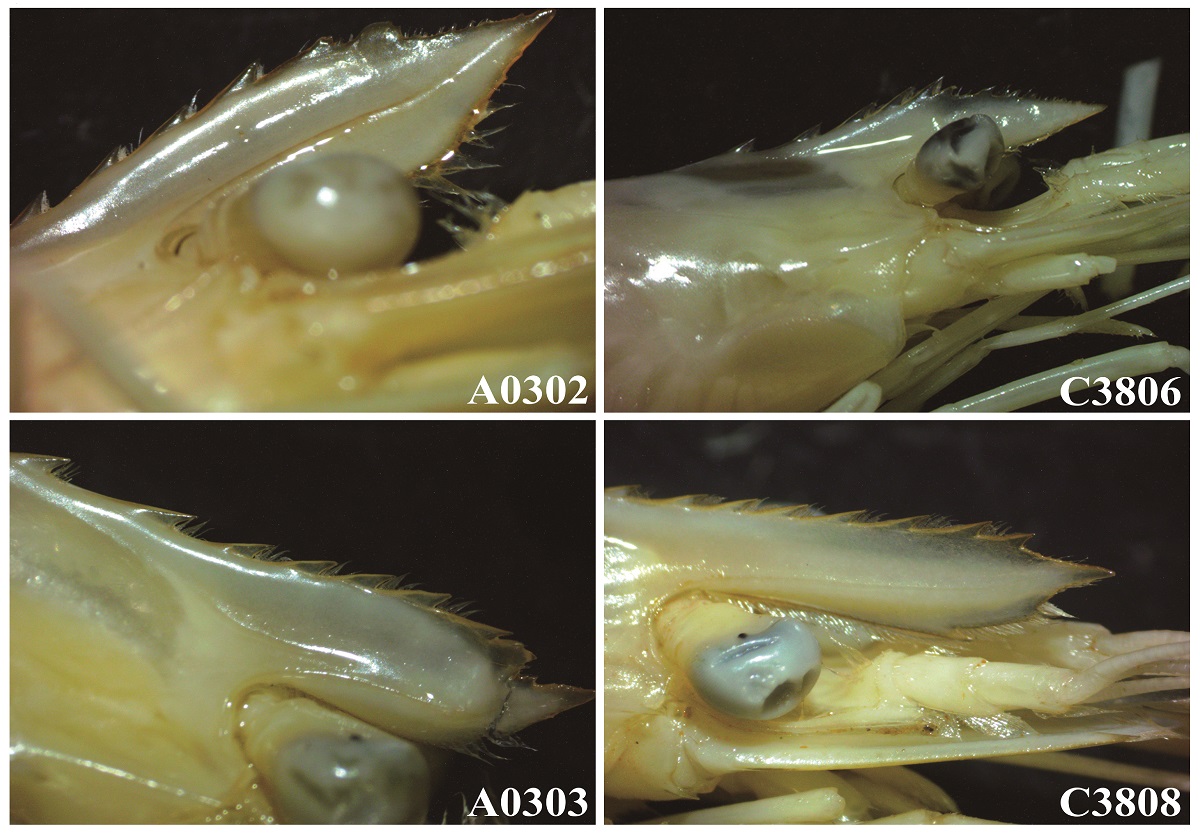

Supplement: Supplementary file 1 — Figure S1 [file ECE3-11-10502-s003.jpg]
